# Herb-symptom analysis of Erchen decoction combined with Xiebai powder formula and its mechanism in the treatment of chronic obstructive pulmonary disease

**DOI:** 10.3389/fphar.2023.1117238

**Published:** 2023-05-19

**Authors:** Hua Ye, Beibei He, Yujie Zhang, Ziwei Yu, Yifan Feng, Chuanbiao Wen, Chongcheng Xi, Quansheng Feng

**Affiliations:** ^1^ School of Intelligent Medicine, Chengdu University of Traditional Chinese Medicine, Chengdu, China; ^2^ School of Pharmacy, Chengdu University of Traditional Chinese Medicine, Chengdu, China; ^3^ Pharmaceutics Center, Institute of Medicinal Plant Development, Chinese Academy of Medical Sciences and Peking Union Medical College, Beijing, China; ^4^ School of Basic Medical Sciences, Chengdu University of Traditional Chinese Medicine, Chengdu, China

**Keywords:** chronic obstructive pulmonary disease, Erchen decoction, Xiebai powder, synergistic mechanism, traditional Chinese medicine, herb-symptom analysis

## Abstract

**Background:** In recent years, the incidence and mortality rates of chronic obstructive pulmonary disease (COPD) have increased significantly. Erchen Decoction combined with Xiebai Powder (ECXB) formula is mainly used to treat lung diseases in traditional Chinese medicine (TCM). However, the active ingredients of ECXB formula, COPD treatment-related molecular targets, and the mechanisms are still unclear. To reveal its underlying action of mechanism, network pharmacology, molecular docking, and molecular dynamic (MD) simulation approaches were used to predict the active ingredients and potential targets of ECXB formula in treating COPD. As a result, Herb-Symptom analysis showed that the symptoms treated by both TCM and modern medicine of ECXB formula were similar to the symptoms of COPD. Network pharmacology identified 170 active ingredients with 137 targets, and 7,002 COPD targets was obtained. 120 targets were obtained by intersection mapping, among which the core targets include MAPK8, ESR1, TP53, MAPK3, JUN, RELA, MAPK1, and AKT1. Functional enrichment analysis suggested that ECXB formula might exert its treat COPD pharmacological effects in multiple biological processes, such as cell proliferation, apoptosis, inflammatory response, and synaptic connections, and ECXB formula treated COPD of the KEGG potential pathways might be associated with the TNF signaling pathway, cAMP signaling pathway, and VEGF signaling pathway. Molecular docking showed that ECXB formula treatment COPD core active ingredients can bind well to core targets. MD simulations showed that the RELA-beta-sitosterol complex and ESR1-stigmasterol complex exhibited higher conformational stability and lower interaction energy, further confirming the role of ECXB formula in the treatment of COPD through these core components and core targets. Our study analyzed the medication rule of ECXB formula in the treatment of COPD from a new perspective and found that the symptoms treated by both TCM and modern medicine of ECXB formula were similar to the symptoms of COPD. ECXB formula could treat COPD through multi-component, multi-target, and multi-pathway synergistic effects, providing a scientific basis for further study on the mechanism of ECXB formula treatment of COPD. It also provides new ideas for drug development.

## 1 Introduction

Chronic obstructive pulmonary disease (COPD) is a common chronic respiratory disease mainly characterized by incompletely reversible airflow restriction. In Europe and the United States, the prevalence of COPD ranges from 3.4% to 13.4% (Blanco et al., 2018), However, in Asia, its prevalence ranges from 3.5% to 19.1% (Buist et al., 2007). Outpatient visit cost accounts for 15%–41% of the total direct cost in the United States and Europe and 4%–48% of the total direct cost in Asian countries (Rehman et al., 2020), one of the main contributors to the global burden of disease. Hence, an in-depth study on COPD is still an urgent need.

Global Strategy for the Diagnosis, Management, and Prevention of Chronic Obstructive Lung Disease: the GOLD science committee report 2019 Singh et al. (2019) proposed stable COPD treatment methods mainly include smoking cessation, bronchodilator, mucolytic agent, immunomodulator, respiratory function exercise, home oxygen therapy, and nutritional therapy. The above methods can improve the symptoms, respiratory function, and quality of life of patients with stable COPD to a certain extent, but all of them need to be maintained for a long time. Due to the high price of non-invasive ventilators, in addition, some patients believe that the comfort of wearing is not good, poor compliance, lack of knowledge, and other factors limit, little benefit, and increase the economic burden for patients.

COPD belongs to the category of “lung distension” in traditional Chinese medicine (TCM), and TCM has unique advantages in the treatment of COPD. Zhen et al. (2018) confirmed in the systematic evaluation and meta-analysis of TCM tonalizing kidney therapy (Bu shen) in the treatment of stable chronic obstructive pulmonary disease that tonalizing kidney therapy can increase lung capacity and reduce the number of CD4^+^ and CD8^+^ lymphocytes in patients with stable chronic obstructive pulmonary disease. Huang et al. (2022) found that the TCM combined with Western medicine (WM) can effectively treat the symptoms related to COPD, the treatment efficiency is significantly improved compared with traditional WM. Therefore, it is very meaningful to study the treatment of COPD with TCM.

Erchen Decoction is composed of Chenpi (*Pericarpium Citri Reticulatae*), Banxia (*Pinellia ternata*), Fulin (*Poria cocos*) and Gancao (*Glycyrrhizae Radix et Rhizome*), Xiebai Powder is composed of Sangbaipi (*Mori cortex*), Digupi (*Lycii Cortex*), Gancao (*Glycyrrhizae radix et rhizome*), Non-glutinous Rice, both TCM formulas have been widely used to treat respiratory diseases (Deng et al., 2020; Zhang et al., 2022). In China, two enduring and effective Chinese classical formulas derived from TCM with a long-standing history spanning centuries, namely, Erchen Decoction combined with Xiebai Powder (ECXB formula), have been extensively employed in treating COPD. Mei and Li (2012) observed the effect of ECXB formula in the treatment of acute exacerbation of COPD and found that the use of this formula can shorten the course of COPD and improve the curative effect. The empirical formula created by famous veteran doctors of TCM is derived from ECXB formula, which can control infection and the accompanied symptoms caused by infection and has good clinical effects in treating COPD (Li and Luo, 2014). So far, the main components, target, and potential synergy mechanism of ECXB formula are not yet clear.

**FIGURE 1:**
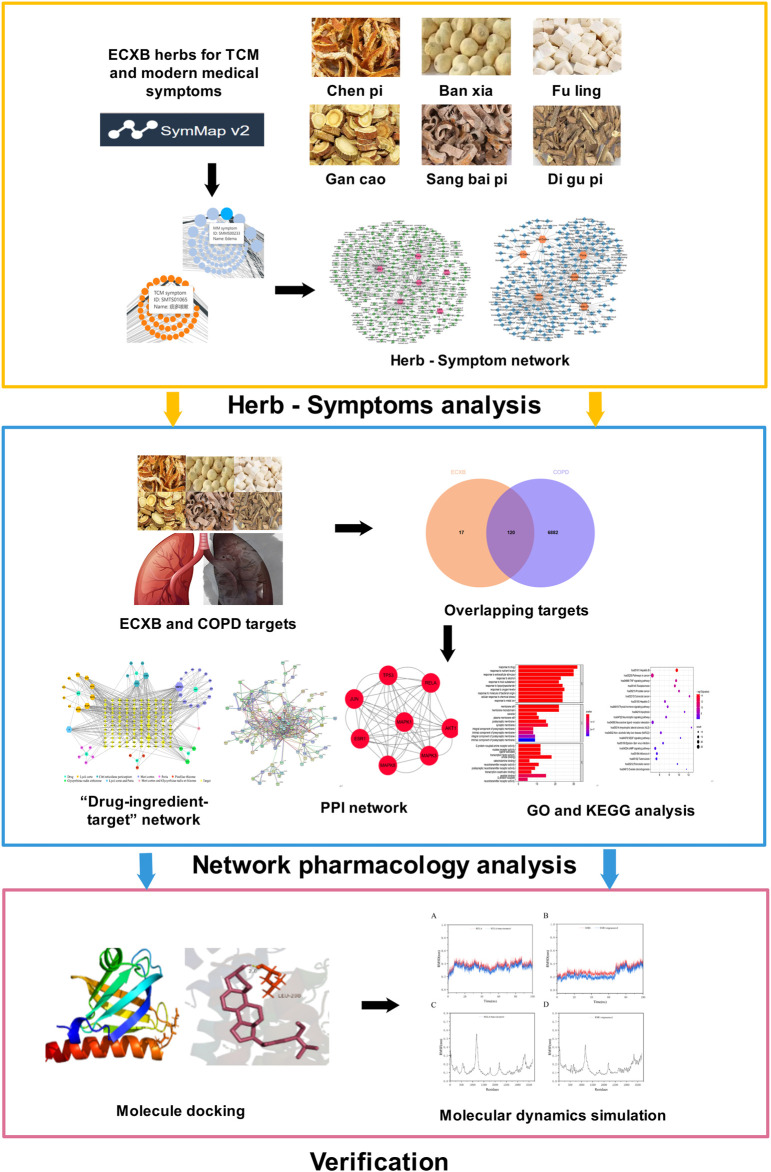
The flowchart of this study is based on Herb-Symptom analysis, network pharmacology, molecular docking, and molecular dynamics simulation for deciphering the potential mechanisms of ECXB formula treatment COPD.

In this study, we aimed to use the SymMap platform to analyze the relationship between the symptoms treated by ECXB formula and the symptoms of COPD. Furthermore, network pharmacology, molecular docking, and molecular dynamic (MD) simulation approaches explore potential targets and mechanisms of ECXB formula for COPD treatment. This study provided a scientific basis for future validation experiments and clinical applications. The detailed flowchart of this study is summarized in Figure 1.

## 2 Materials and methods

### 2.1 “Herb-symptom” network construction

SymMap platform (http://www.symmap.org/) (Wu et al., 2019) was utilized to collect related symptoms treated by both TCM and modern medicine of *Pinellia ternata, Pericarpium Citri Reticulatae, Poria cocos, Glycyrrhizae Radix et Rhizome, Lycii cortex*, and *Mori cortex* in the formula of ECXB. Cytoscape 3.9.0 software (Shannon et al., 2003) was used for visual analyses of the network of “Herb-TCM symptoms” and “Herb-modern medicine symptoms.” According to the International Clinical Practice Guideline of Chinese Medicine Chronic Obstructive Pulmonary Disease (Li, 2020) and Global Strategy for the Diagnosis, Management, and Prevention of Chronic Obstructive Lung Disease: the GOLD science committee report 2019 (Singh et al., 2019), related COPD symptoms were collected.

### 2.2 Collection of active ingredients and prediction on targets of ECXB formula

The active ingredients of ECXB formula were searched by the TCM Systems Pharmacology Database and Analysis Platform (TCMSP, https://old.tcmsp-e.com/tcmsp.php) (Ru et al., 2014). Oral bioavailability (OB) (Xu et al., 2012) represents the percentage of an orally administered dose of an unchanged drug that reaches the systemic circulation, which reveals the convergence of the ADME process. High oral bioavailability is often a key indicator to determine the drug-like property of bioactive molecules as therapeutic agents. Drug-likeness (DL) (Tao et al., 2013) is a qualitative concept used in drug design to estimate how “drug-like” a prospective compound is, which helps to optimize pharmacokinetic and pharmaceutical properties, such as solubility and chemical stability. The “drug-like” level of the compounds is 0.18, which is used as a selection criterion for the “drug-like” compounds in traditional Chinese herbs. Therefore, the active ingredients were screened according to the following criteria: OB ≥ 30% and DL ≥ 0.18. Using the PubChem database (https://pubchem.ncbi.nlm.nih.gov/) (Kim et al., 2021), the SMILE number corresponding to the active ingredients screened from the TCMSP database was downloaded. The SMILE structure of active ingredients was uploaded to the Swiss Target Prediction database (http://www.swisstargetprediction.ch/) (Daina et al., 2019) for target prediction. The predicted targets of active ingredients were obtained and integrated. UniProt database (https://www.uniprot.org/) (Bateman et al., 2021) was used for standardized and unified processing of target names, and the species was limited to “HUMAN.”

### 2.3 Collection of disease target and prediction of intersection target

By taking “Chronic obstructive pulmonary diseases” as the keyword, the related targets were searched in the GeneCards^®^ database (https://www.genecards.org) (Safran et al., 2021) and OMIM ^®^ database (https://omim.org/) (Amberger et al., 2015). The predicted targets of ECXB formula were mapped to the related targets of COPD, and the common targets of ECXB formula in the treatment of COPD were obtained and further visualized using a Venn diagram.

### 2.4 “Drug-ingredient-target” network and core active ingredients screening

The common targets of ECXB formula in the treatment of COPD have imported into Cytoscape 3.9.0 software, the network diagram of “Drug-ingredient-target” was constructed, and the core active ingredients were screened according to the degree value ranking.

### 2.5 PPI network construction and screening of core protein targets

The intersection target of ECXB formula in the treatment of COPD was imported into the STRING online website (https://cn.string-db.org/) (Szklarczyk et al., 2019), and the protein type was set as “*Homo sapiens*”, the highest confidence parameter score was greater than 0.7, and other parameters were set as default values (network type: full STRING network, meaning of network edges: evidence). The core network of ECXB formula for the treatment of COPD protein interaction was constructed, and the TSV file was exported. Node1, node2, and the combined score in the TSV file were imported into Cytoscape 3.9.0 software, mapping interaction networks, and screening core protein targets.

### 2.6 Enrichment analysis of GO and KEGG

Gene Ontology (GO) analysis and Kyoto Encyclopedia of Gene and Genome (KEGG) pathway analysis for ECXB formula in the treatment of COPD targets were conducted by using the DAVID database (https://david.ncifcrf.gov/) (Huang et al., 2009b; Sherman et al., 2022). The results of the two analysis methods were mapped in the form of histograms and bubbles by using Bioinformatics online platform (http://www.bioinformatics.com.cn/) (Huang et al., 2009a).

### 2.7 Molecular docking verification

In the PDB database (https://www.rcsb.org/) (Berman, 2000), the crystal structure of the core target protein was selected and its “PDB” format file was downloaded. The core active ingredients in the “2D SDF” structure were downloaded from the PubChem database and imported into Chem3D 18.0 for their optimization. The PDB files of ligand molecules were imported into AutoDock Tools for processing, and saved as a PDBQT format file for later use. AutoDock 4.2 was used to dock the processed active ingredients with the core target protein (Morris et al., 2009). Using the minimum binding energy as the docking result of the target protein and ligand, the Pymol 2.5.2 software (https://pymol.org/2/) was used for observation and mapping.

### 2.8 Molecular dynamics simulation

GROMACS (2020.3) (Van Der Spoel et al., 2005) was used to analyze the MD simulation to check the stability of the protein-ligand complexes. The docking models of the complexes on the top two molecular docking results were used as the initial conformation for MD simulations. The root mean square deviation (RMSD) of each complex was analyzed to measure the stability of the complex system according to the degree of the molecular structure change. The root mean square fluctuation (RMSF) of the identified complexes was analyzed to understand the relative fluctuation of proteins. The receptor-ligand binding free energy was calculated using the Molecular Mechanics Poisson−Boltzmann Surface Area (MMPBSA) method in the 25 ns MD simulation trajectory.

## 3 Results

### 3.1 Analysis of “herb-symptom” network

**FIGURE 2:**
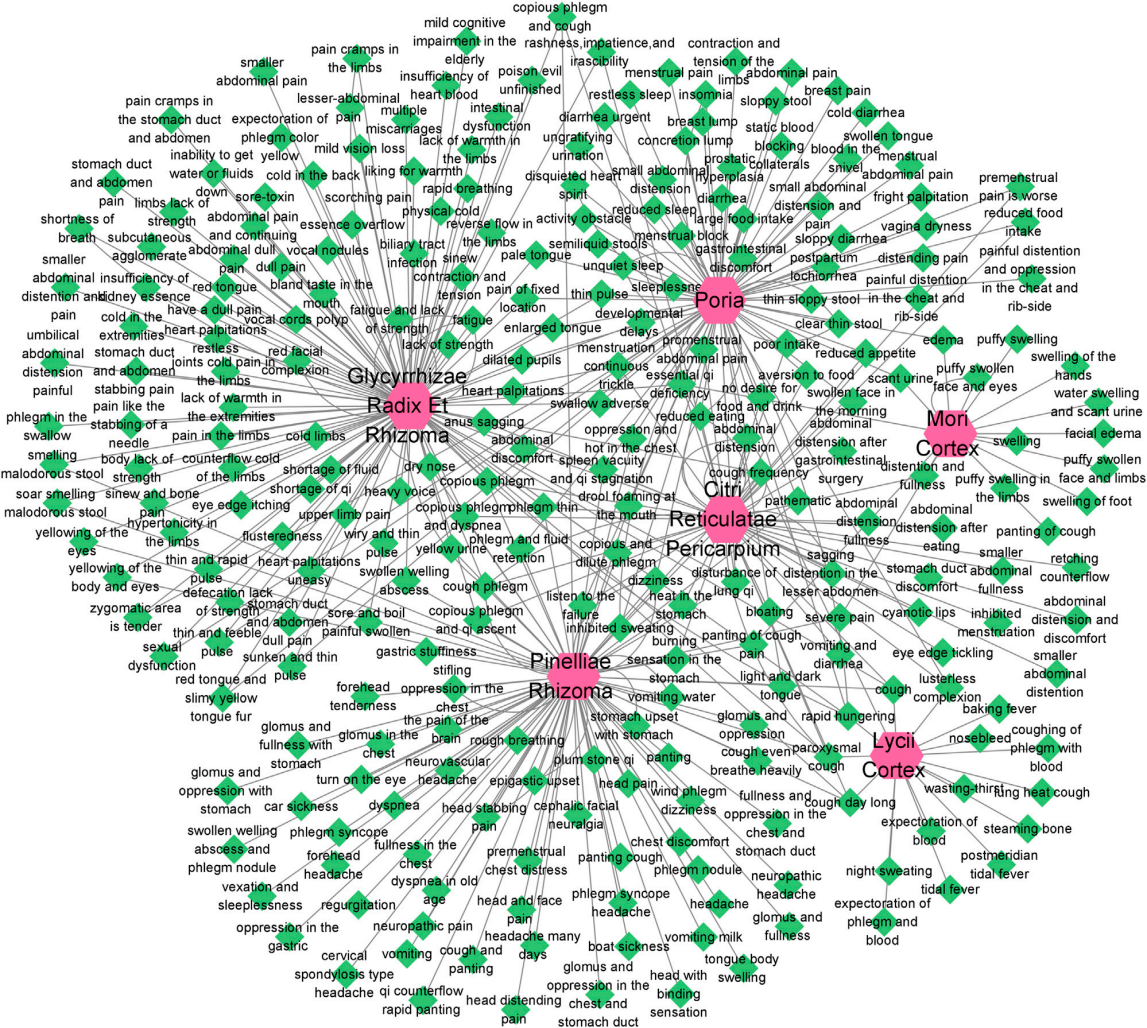
“Herb-TCM Symptom” network diagram. The pink node represents herbs, while green nodes show the TCM Symptoms.

**FIGURE 3:**
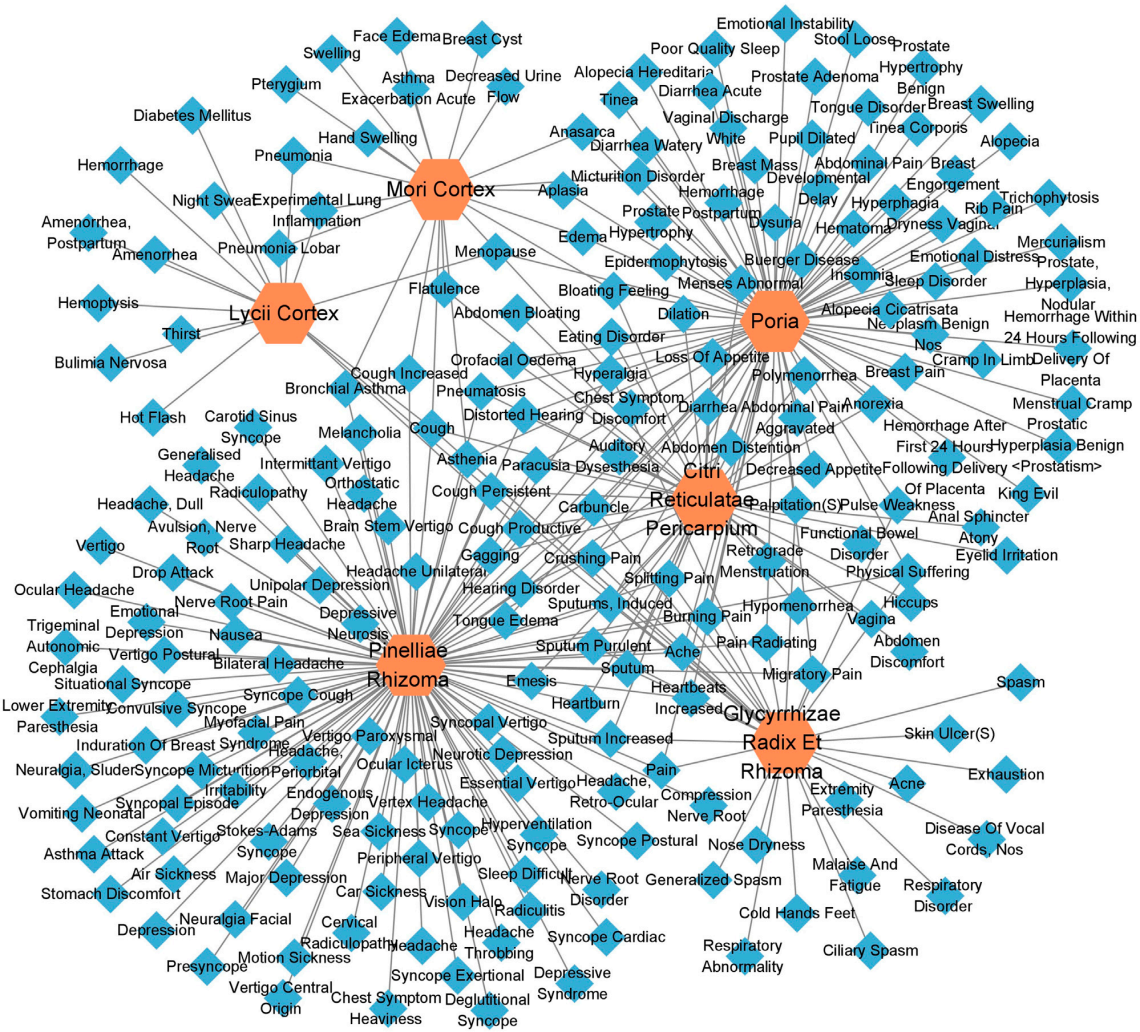
“Herb-modern medicine Symptom” network diagram. The orange node represents herbs, while the blue nodes show modern medicine Symptoms.

According to the information related to symptoms treated by both TCM and modern medicine of diseases treated by herbs in the formula of ECXB collected on the SymMap platform, a total of 258 TCM symptoms were obtained, including 93 symptoms treated by *Pinelliae rhizome,* 54 by *Citri reticulatae pericarpium*, 84 by *Poria*, 116 by *Glycyrrhizae radix et rhizome,* 16 by *Lycii cortex*, and 16 by *Mori cortex*, as shown in Figure 2 (Supplementary Table S1). A total of 207 symptoms were treated by modern medicine, including 104 by *Pinelliae rhizome*, 37 by *Citri reticulatae pericarpium*, 81 by Poria, 33 by *Glycyrrhizae radix et rhizome*, 16 by *Lycii cortex*, and 21 by Mori cortex, as shown in Figure 3 (Supplementary Table S2). Symptoms targeted by TCM and modern medicine as well as COPD symptoms are shown in Table 1. It was found that the symptoms treated by both TCM and modern medicine of ECXB formula were the same as those of COPD patients with dyspnea, coughing up phlegm, chronic cough, and chest breathing, indicated that ECXB formula treatment for COPD is consistent with symptomatic treatment of TCM.

**TABLE 1:** Symptoms targeted by TCM and modern medicine as well as COPD symptoms.

Herb	Symptoms treated by TCM	Symptoms treated by modern medicine	COPD symptoms
*Pinelliae rhizome*	Cough, panting, stifling oppression in the chest	Cough, Sputum, Hyperventilation Syncope	Dyspnea
*Poria*	The lusterless complexion, edema, phlegm, and fluid retention	Tongue Edema, Anasarca, Sputum	Coughing up phlegm
*Glycyrrhizae radix et rhizome*	cough phlegm, fatigue and lack of strength, lack of warmth in the limbs	Cough Persistent, Sputum, Malaise and Fatigue, Respiratory Disorder, Extremity Paresthesia	Chronic cough
*Citri reticulatae pericarpium*	cough phlegm, reduced eating	Sputum Increased, Loss Of Appetite	Loss of appetite
*Mori cortex*	panting of cough, puffy swollen face and limbs, scant urine	Asthma Exacerbation Acute, Bronchial Asthma, Face Edema	Chest breathing
*Lycii cortex*	tidal fever, night sweating, lung heat cough, expectoration of phlegm and blood	Pneumonia, Night Sweat, Hot Flash, Cough Persistent	Other symptoms

### 3.2 Acquisition of active ingredients and predicted targets

According to the screening procedure, a total of 1,314 active ingredients of ECXB formula were obtained from TCMSP, including 386 in Pinelliae rhizome, 147 in Citri reticulatae pericarpium, 69 in Poria, 75 in Glycyrrhizae radix et rhizome, 239 in Lycii cortex, and 398 in Mori cortex. Guiding by the ADME and DL standard (OB threshold ≥30% and DL threshold ≥0.18), 170 active ingredients were obtained after removing duplicated compounds. Among them, 13 were extracted from Pinelliae rhizome, 5 in Citri reticulatae pericarpium, 15 in Poria, 92 in Glycyrrhizae radix et rhizome, 13 in Lycii cortex, and 32 in Mori cortex (Supplementary Table S3). Partial results are listed in Table 2. 137 prediction targets of ECXB formula were obtained from the Swiss Target Prediction platform.

**TABLE 2:** Basic information on some active ingredients of ECXB formula.

Herb	MOL Id	Ingredients	OB (%)	DL
*Pinelliae rhizome*	MOL001755	24-Ethylcholest-4-en-3-one	36.08	0.76
	MOL002670	Cavidine	35.64	0.81
	MOL002714	baicalein	33.52	0.21
	MOL002776	Baicalin	40.12	0.75
*Citri reticulatae*	MOL000359	sitosterol	36.91	0.75
*pericarpium*	MOL004328	naringenin	59.29	0.21
*Poria*	MOL000290	Poricoic acid A	30.61	0.76
	MOL000291	Poricoic acid B	30.52	0.75
	MOL000276	7,9(11)-dehydropachymic acid	35.11	0.81
	MOL000289	pachymic acid	33.63	0.81
*Glycyrrhizae*	MOL004835	Glypallichalcone	61.6	0.19
*radix et rhizome*	MOL004841	Licochalcone B	76.76	0.19
	MOL004996	icos-5-enoic acid	30.7	0.2
*Lycii cortex*	MOL001552	OIN	45.97	0.19
	MOL001645	Linoleyl acetate	42.1	0.2
	MOL001689	acacetin	34.97	0.24
*Mori cortex*	MOL012692	kuwanon D	31.09	0.8
	MOL012689	beta-sitosterol	36.79	0.87
	MOL000358	sanggenone H	36.91	0.75
	MOL012755	campest-5-en-3beta-ol	37.5	0.53
	MOL005043	sanguinarine	37.58	0.71
	MOL001474	moracin M-6,3′-di-O-β-D-glucopyranoside	37.81	0.86

(Erchen Decoction is composed of P*inelliae rhizome, Citri reticulatae pericarpium, Poria, and Glycyrrhizae radix et rhizome*. Xiebai Powder is composed of Glycyrrhizae radix et rhizome, Lycii cortex, and Mori cortex.).

### 3.3 Acquisition of targets for COPD and prediction of intersection target

**FIGURE 4:**
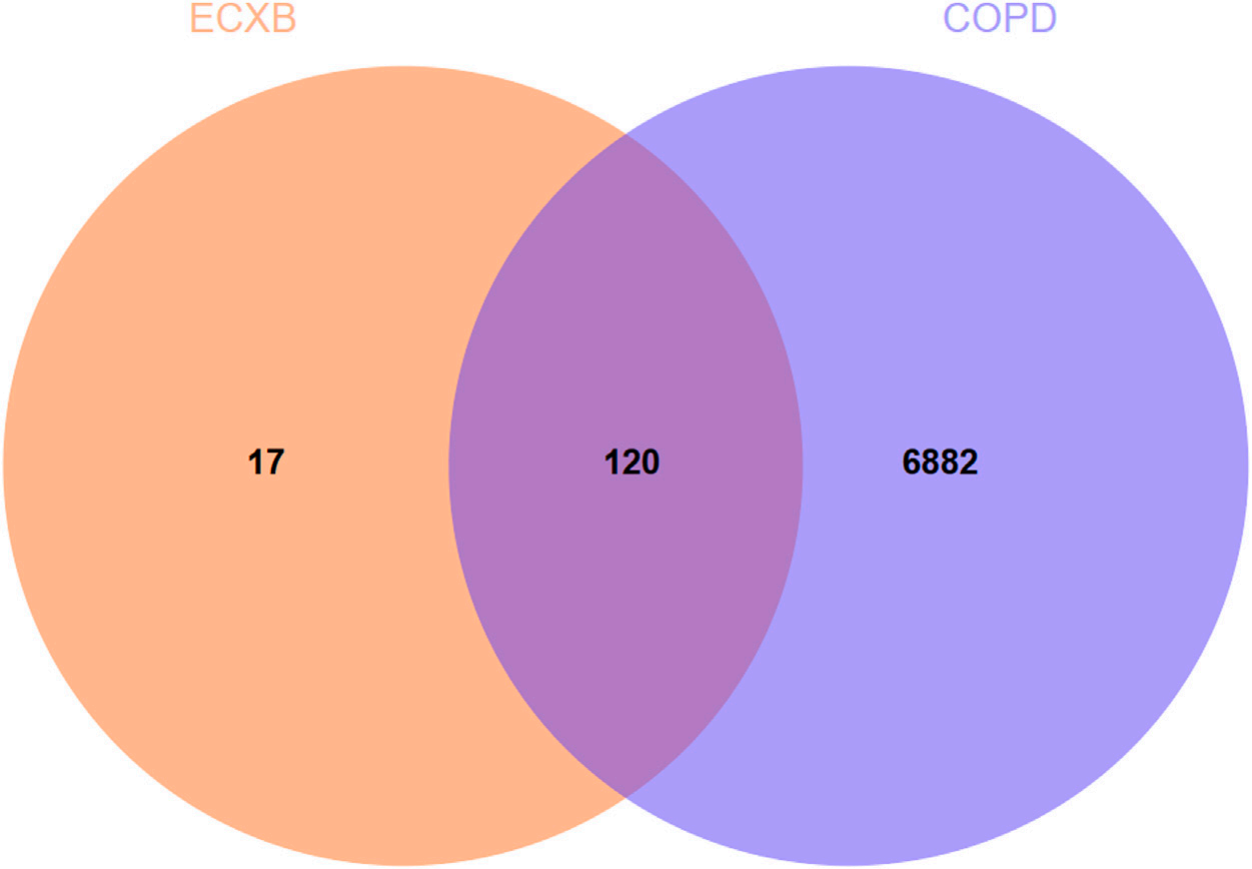
Venn diagram of ECXB formula combined with COPD. The Venn map showed that 120 targets were shared between ECXB formula and COPD.

A total of 7002 COPD-related targets were collected from GeneCards^®^ and OMIM ^®^ databases (Supplementary Table S4). The predicted targets of ECXB formula were intersected with COPD-related targets, then 120 potential targets of ECXB formula for the treatment of COPD were obtained, and Venn diagrams were drawn in Figure 4 (Supplementary Table S5).

### 3.4 Analysis of the “drug-ingredient-target” network and screening of core active ingredients

**FIGURE 5:**
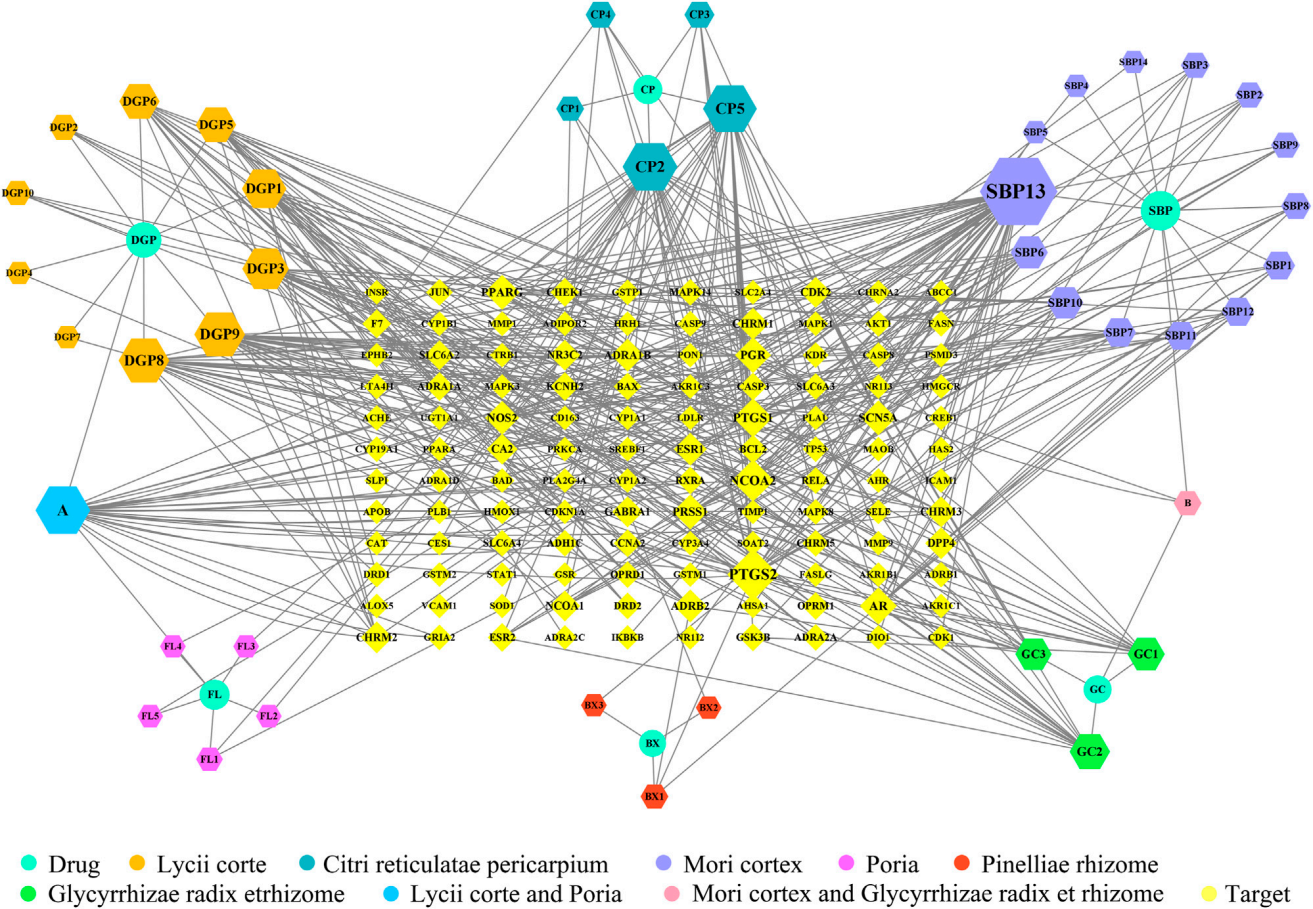
“Drug-ingredient-target” network diagram of ECXB formula in the treatment of COPD. The node size and node label size are determined by the node degree value. The higher the degree value, the larger will be the node and label.

The 120 overlapping targets of ECXB formula and COPD as well as these targets corresponding ingredients were imported into Cytoscape software to construct a “Drug-ingredient-target” network. As shown in Figure 5, nodes in the network diagram represented drugs, active ingredients, and targets. The edges indicated that the nodes can interact with each other. The top 10 active ingredients of degree value were selected as the core ingredients, including kaempferol, hederagenin, naringenin, nobiletin, beta-sitosterol, stigmasterol, acacetin, OIN, atropine, and sugiolas (Table 3).

**TABLE 3:** Core Ingredients of ECXB formula.

Molid	Ingredients	Degree	Number	Drug
MOL000422	kaempferol	52	51	*Mori cortex*
MOL000296	hederagenin	30	14	*Poria, Lycii cortex*
MOL004328	naringenin	30	29	*Citri reticulatae pericarpium*
MOL005828	nobiletin	29	28	*Citri reticulatae pericarpium*
MOL000358	beta-sitosterol	26	25	*Lycii cortex*
MOL000449	Stigmasterol	26	25	*Lycii cortex*
MOL001689	acacetin	22	21	*Lycii cortex*
MOL001552	OIN	20	19	*Lycii cortex*
MOL002219	Atropine	16	15	*Lycii cortex*
MOL002222	sugiol	16	15	*Lycii cortex*

### 3.5 Analysis of PPI network and screening of core targets

**FIGURE 6:**
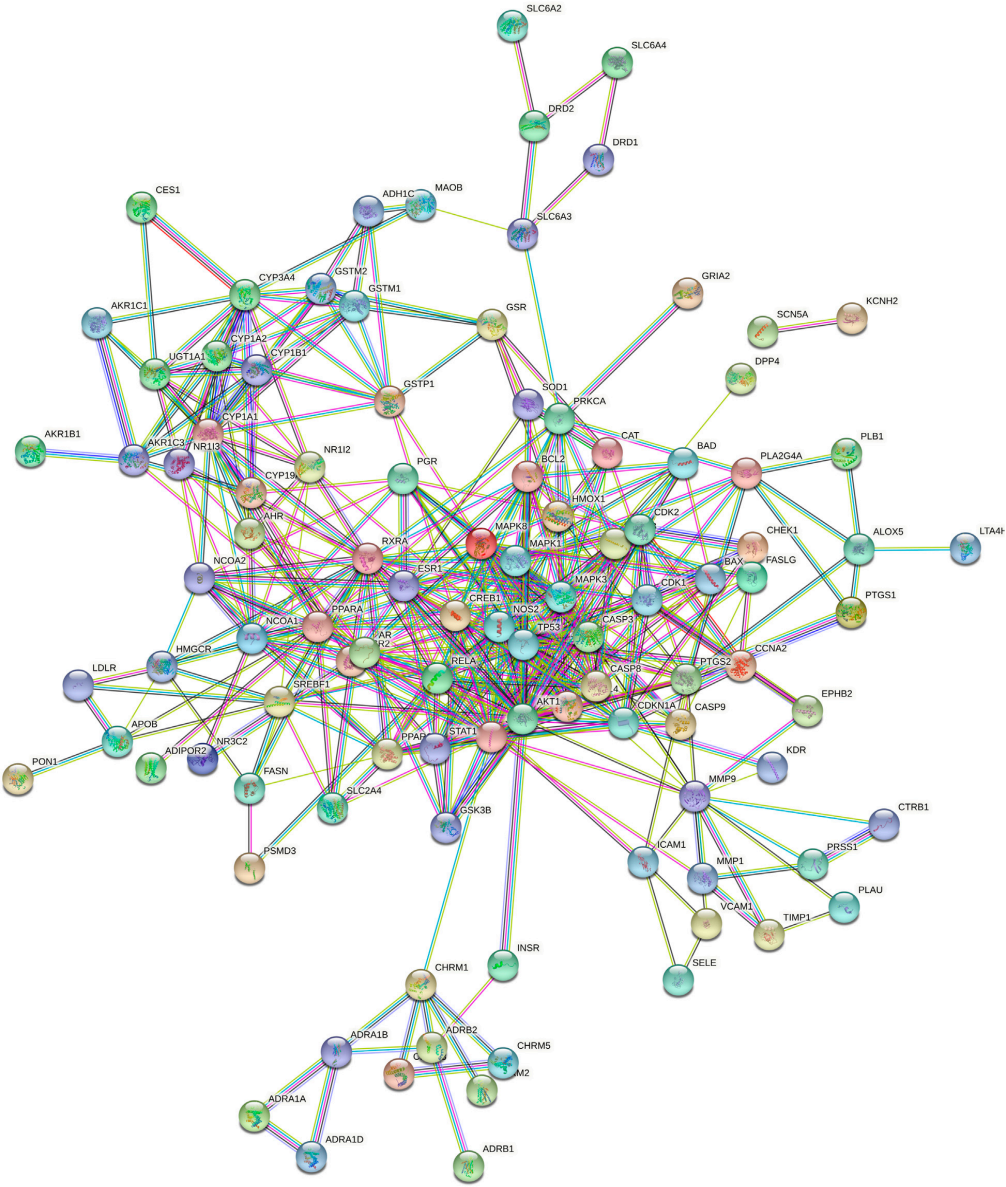
Protein interaction network diagram. 120-node and 424-edge PPI network of potential targets of the ECXB formula working on COPD was acquired at the String database.

**FIGURE 7:**
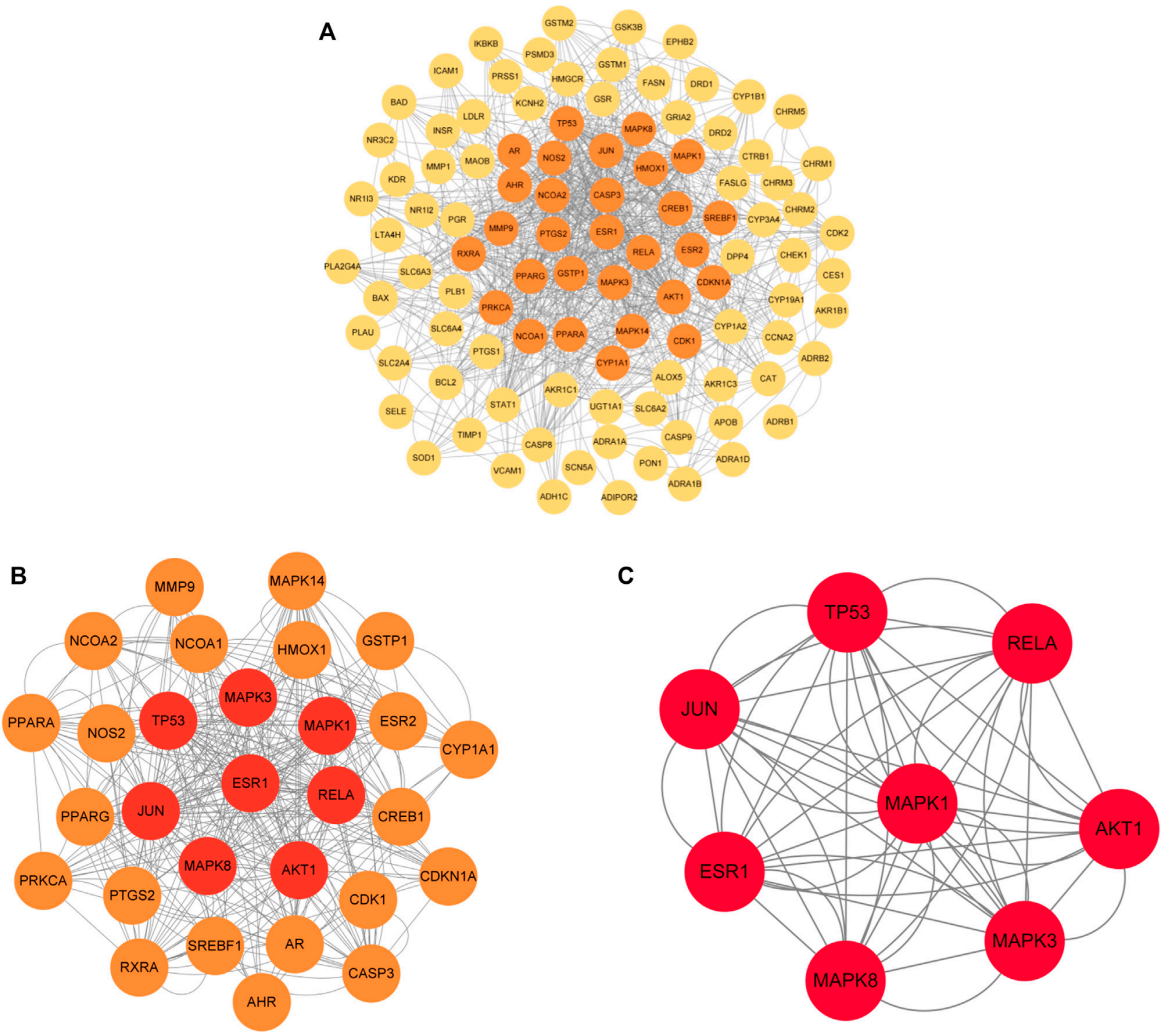
Core protein interaction network. **(A)** Interactive PPI network of ECXB formula-related targets and COPD-related targets. **(B)** PPI network of significant proteins extracted from **(A)**. **(C)** PPI network of candidate COPD targets for ECXB formula treatment extracted from **(B)**.

The 120 intersecting targets were imported into the STRING database, and the obtained information was imported into Cytoscape software to construct a PPI network between ECXB formula and the treatment of COPD, as shown in Figure 6. Using Cytoscape CytoNCA, under the condition that the values of DC, BC, CC, EC, LAC, and NC were all greater than their corresponding median values (Supplementary Tables S6–S8). Among them, 8 core targets were screened, including MAPK8, ESR1, TP53, MAPK3, JUN, RELA, MAPK1, and AKT1, as shown in Figure 7.

### 3.6 Analysis of GO and KEGG pathway

**FIGURE 8:**
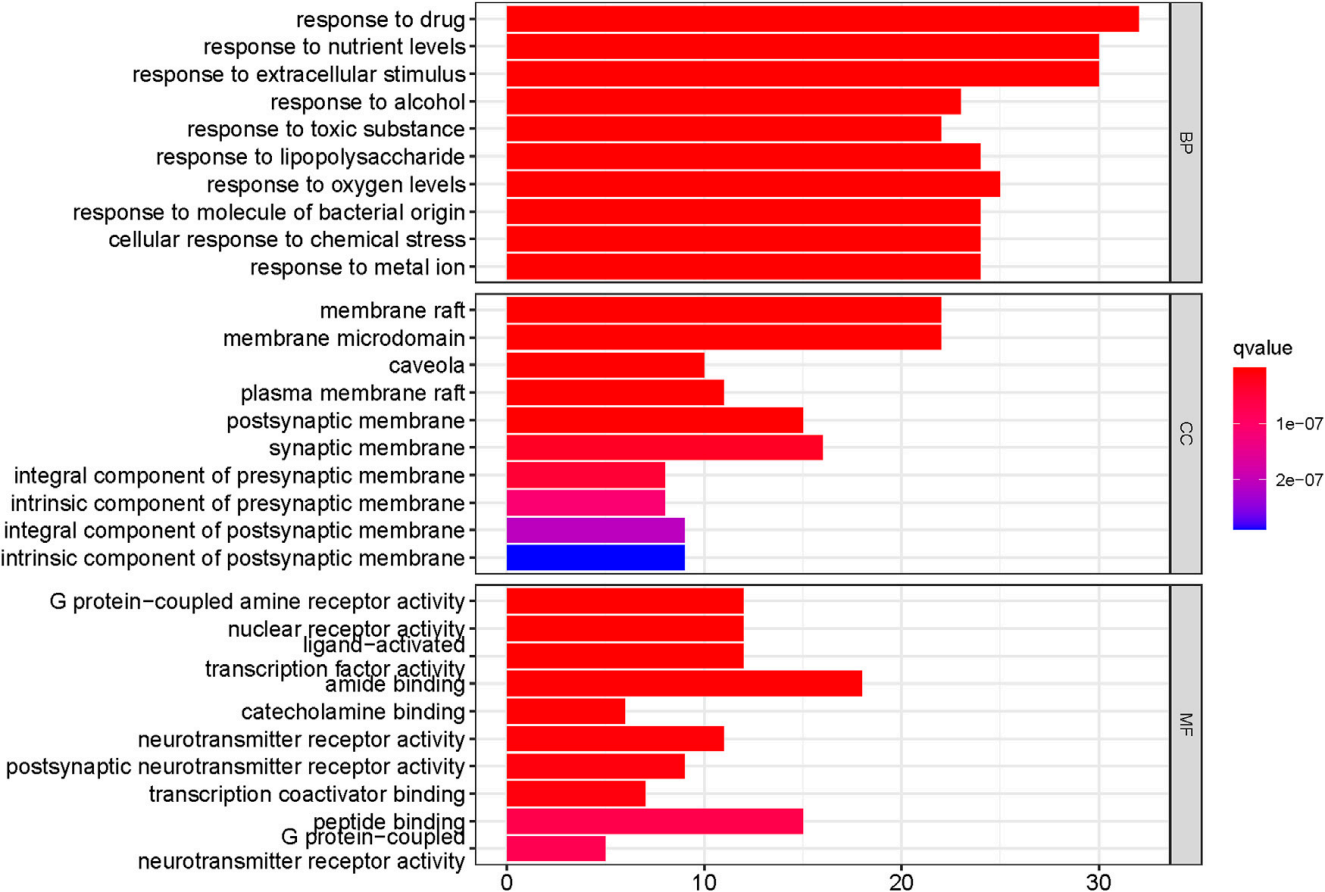
Functional enrichment analysis of the top 10 in GO. The Y-axis shows the enriched GO items of target genes. The X-axis shows the gene counts for the items. The color indicates the q value.

The intersecting targets of ECXB formula for the treatment of COPD were imported into the DAVID database to screen GO entries and KEGG signaling pathways of *p < 0.01*. GO functional enrichment analysis was performed under the conditions of Count ≥ 4, *p* ≤ 0.01, FDR (False Discovery Rate) ≤0.01, and a total of 563 results were obtained. There were 403 Biological Processes (BPs), 109 Molecular Functions (MFs), and 51 Cellular Components (CCs) (Supplementary Table S9). The top 10 results of count values were selected to draw a histogram, as shown in Figure 8.

**FIGURE 9:**
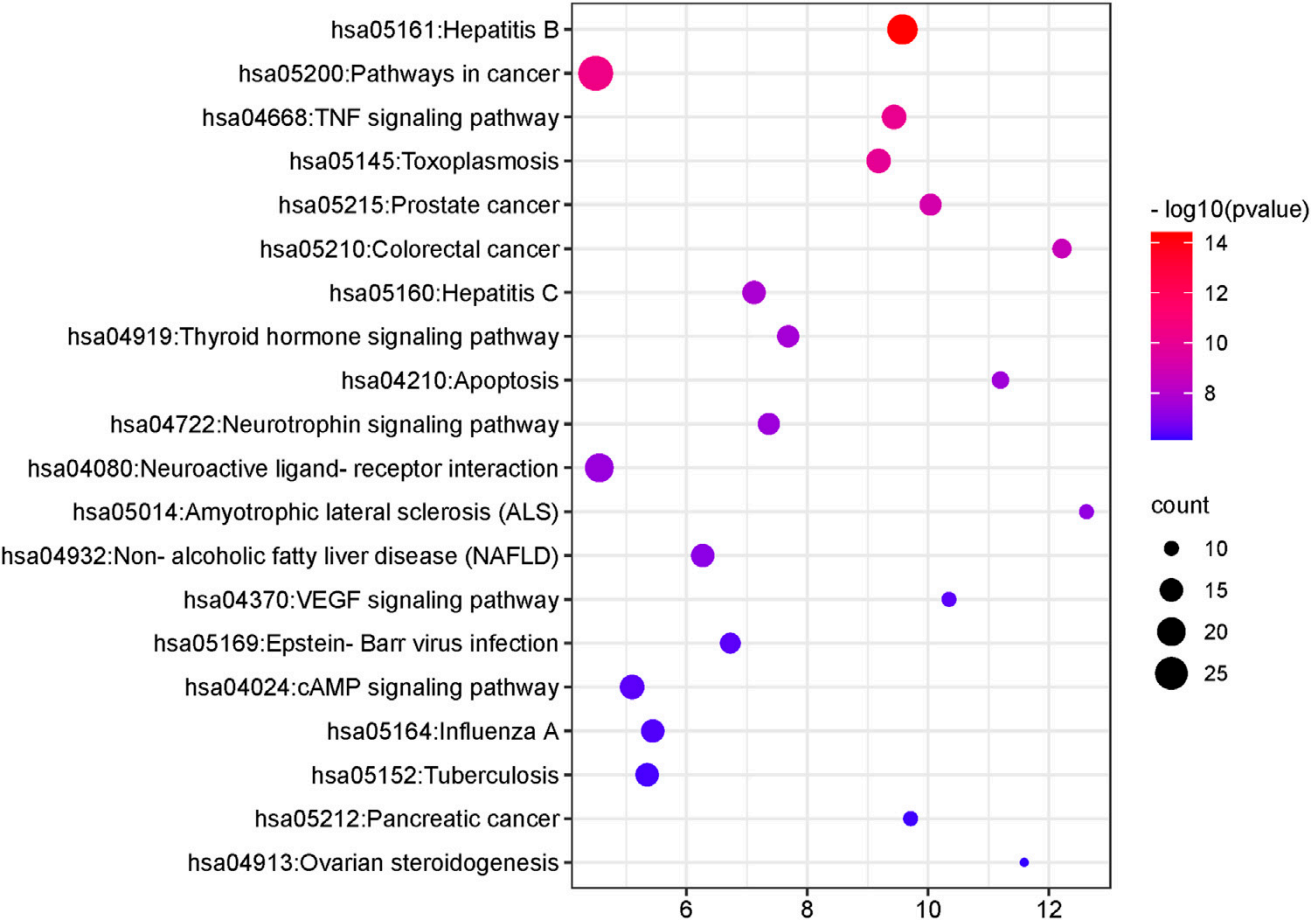
The first 20 KEGG pathways. The Y-axis shows the enriched KEGG pathways for target genes. The X-axis shows the gene counts for the items. The color indicates the *p*-value.

**FIGURE 10:**
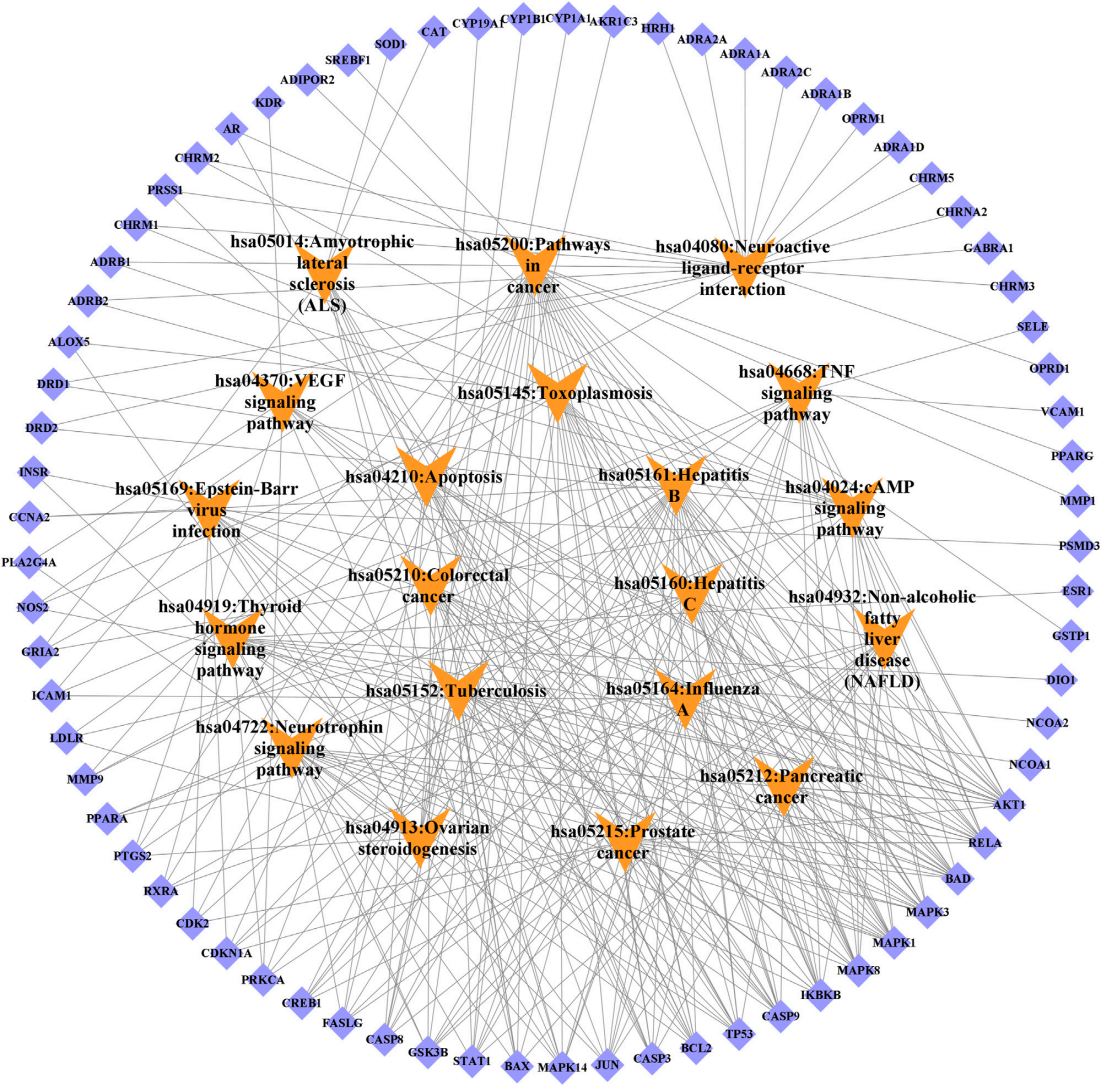
Target-signal pathway network diagram. The orange boxes indicate the signal pathways. The blue boxes indicate the gene names.

**FIGURE 11:**
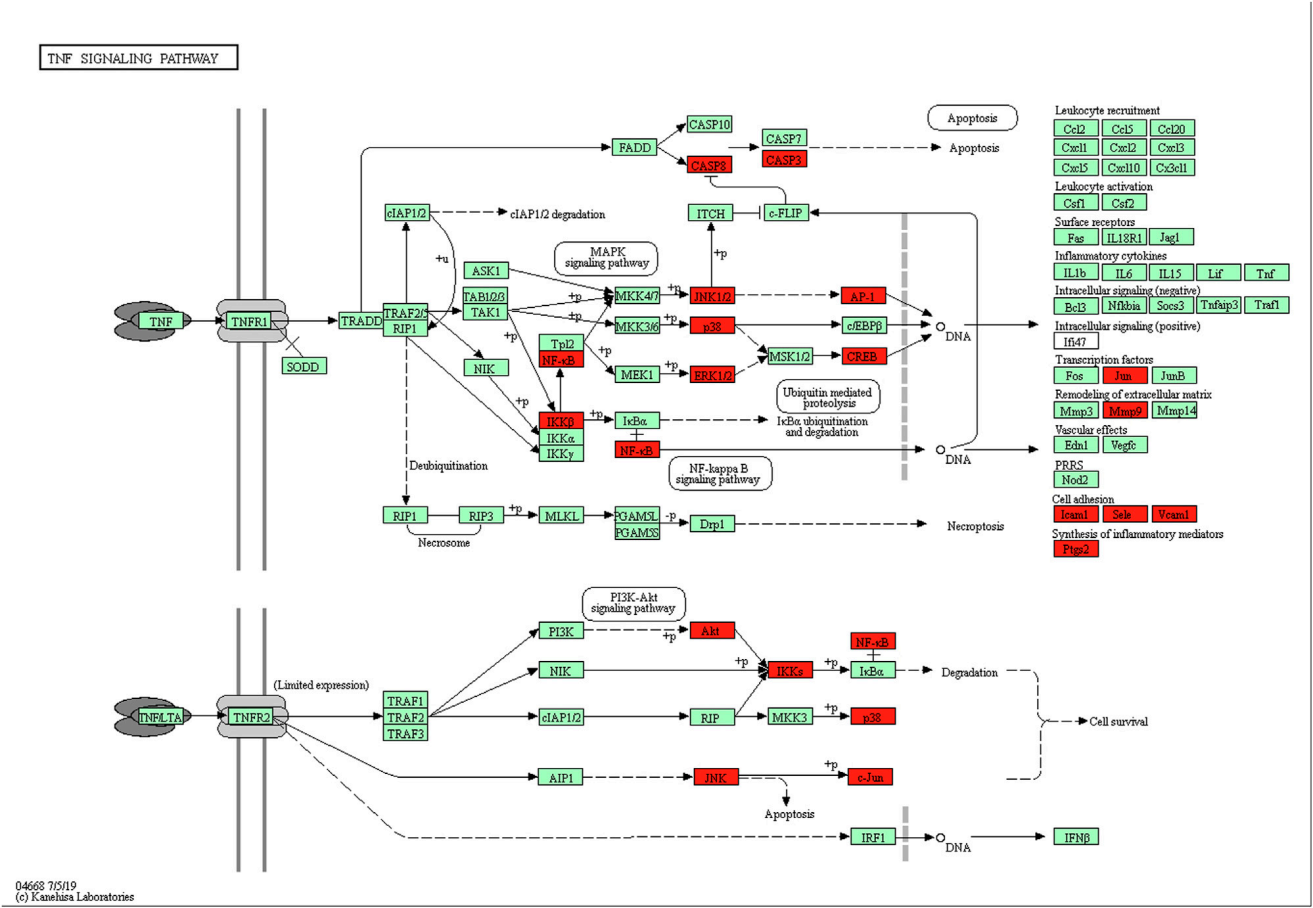
The TNF-α signaling pathway of potential target genes of ECXB formula in COPD. Arrows indicate upstream and downstream relationships between genes. The red blocks are ECXB formula target genes in the network.

KEGG pathway analysis showed that the intersecting targets mapped 87 KEGG signaling pathways (Supplementary Table S10), and the top 20 pathways were selected to draw a bubble map, as shown in Figure 9. Detailed data are shown in Table 4. The targets and 20 pathways were imported into Cytoscape to construct a “target-pathway” network (Figure 10). Therefore, the network analysis suggested that ECXB formula may play a therapeutic role in COPD treatment by regulating signaling pathways, including the TNF signaling pathway, cAMP signaling pathway, VEGF signaling pathway, Hepatitis B Pathways, and Hepatitis C Pathways, suggesting that these pathways may mediate the treatment of COPD. Among them, the TNF signaling pathway was the KEGG pathway with a large number of gene enrichment and obvious significance. The visualization analysis of the TNF signaling pathway is shown in Figure 11.

**TABLE 4:** KEGG pathway analysis for the treatment of COPD.

ID	Pathway	*p*-value	Count
hsa05417	Lipid and atherosclerosis	4.70E-20	28
hsa05207	Chemical carcinogenesis - receptor activation	5.73E-18	26
hsa05161	Hepatitis B	2.18E-17	23
hsa05215	Prostate cancer	1.26E-14	17
hsa01524	Platinum drug resistance	4.25E-14	15
hsa05160	Hepatitis C	3.44E-13	19
hsa04668	TNF signaling pathway	2.19E-12	16
hsa05145	Toxoplasmosis	2.19E-12	16
hsa04933	AGE-RAGE signaling pathway in diabetic complications	5.43E-12	15
hsa05418	Fluid shear stress and atherosclerosis	5.67E-12	17
hsa05167	Kaposi sarcoma-associated herpesvirus infection	1.58E-11	19
hsa05208	Chemical carcinogenesis - reactive oxygen species	2.22E-11	20
hsa04932	Non-alcoholic fatty liver disease	3.36E-11	17
hsa01522	Endocrine resistance	5.74E-11	14
hsa05162	Measles	6.40E-11	16
hsa05210	Colorectal cancer	1.40E-10	13
hsa05169	Epstein-Barr virus infection	2.76E-10	18
hsa05222	Small cell lung cancer	3.35E-10	13
hsa04657	IL-17 signaling pathway	4.42E-10	13
hsa05212	Pancreatic cancer	4.43E-10	12
hsa04210	Apoptosis	4.89E-10	15
hsa05170	Human immunodeficiency virus 1 infection	6.10E-10	18
hsa04722	Neurotrophin signaling pathway	8.17E-10	14
hsa04919	Thyroid hormone signaling pathway	1.02E-09	14
hsa04370	VEGF signaling pathway	6.63E-09	10
hsa04936	Alcoholic liver disease	8.48E-09	14
hsa05163	Human cytomegalovirus infection	1.14E-08	17
hsa05152	Tuberculosis	2.41E-08	15
hsa05022	Pathways of neurodegeneration - multiple diseases	2.64E-08	24
hsa04913	Ovarian steroidogenesis	2.71E-08	9

### 3.7 Analysis of molecular docking

**FIGURE 12:**
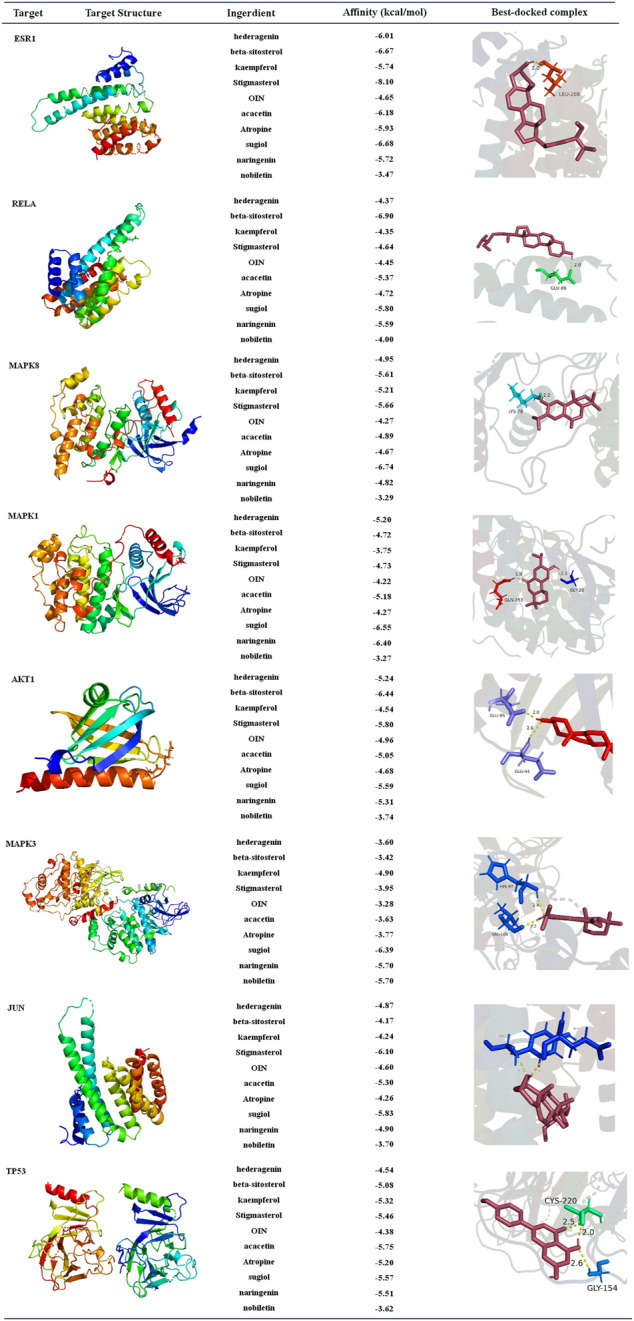
Diagram of molecular docking results. Proteins are shown to interact with molecules. The docking scores are shown in the middle. The binding amino acidic sites and other details are shown on the right.

The selected top ten active ingredients included hederagenin (MOL000296), beta-sitosterol (MOL000358), kaempferol (MOL000422), Stigmasterol (MOL000449), OIN (MOL001552), acacetin (MOL001689), Atropine (MOL002219), surgical (MOL002222), naringenin (MOL004328), and nobiletin (MOL005828). The protein structures of core targets were acquired online from RCSB PDB, including MAPK8 (PDB ID: 2XRW), TP53 (PDB ID: 6GGC), MAPK3 (PDB ID: 4QTB), MAPK1 (PDB ID: 4ZZN), AKT1 (PDB ID: 1unq), ESR1 (PDB ID: 7baa), JUN (PDB ID: 6y3v), and RELA (PDB ID: 6nv2). The molecular docking for the top ten active ingredients and the protein structures of core targets were conducted by using AutoDock 4.2. The results of molecular docking indicate that the lower the binding energy, the more stable the active ingredients and targets are (Supplementary Table S11). Processed by PyMOL software, the optimal docking image of the active ingredients and the targets was displayed. It can be seen from Figure 12 that the best affinity modes were RELA-beta-sitosterol and ESR1-stigmasterol. Table 5.

**TABLE 5:** The binding free energy and energy components of MD simulation.

Energy	RELA _ beta-sitosterol	ESR1 _ stigmasterol
Van der Waals Energy (KJ/mol)	−144.687	−135.674
Electrostatic Energy (kJ/mol)	−1.605	1.243
Total Binding Energy (KJ/mol)	−129.870	−121.686

### 3.8 Results of molecular dynamics simulation

**FIGURE 13:**
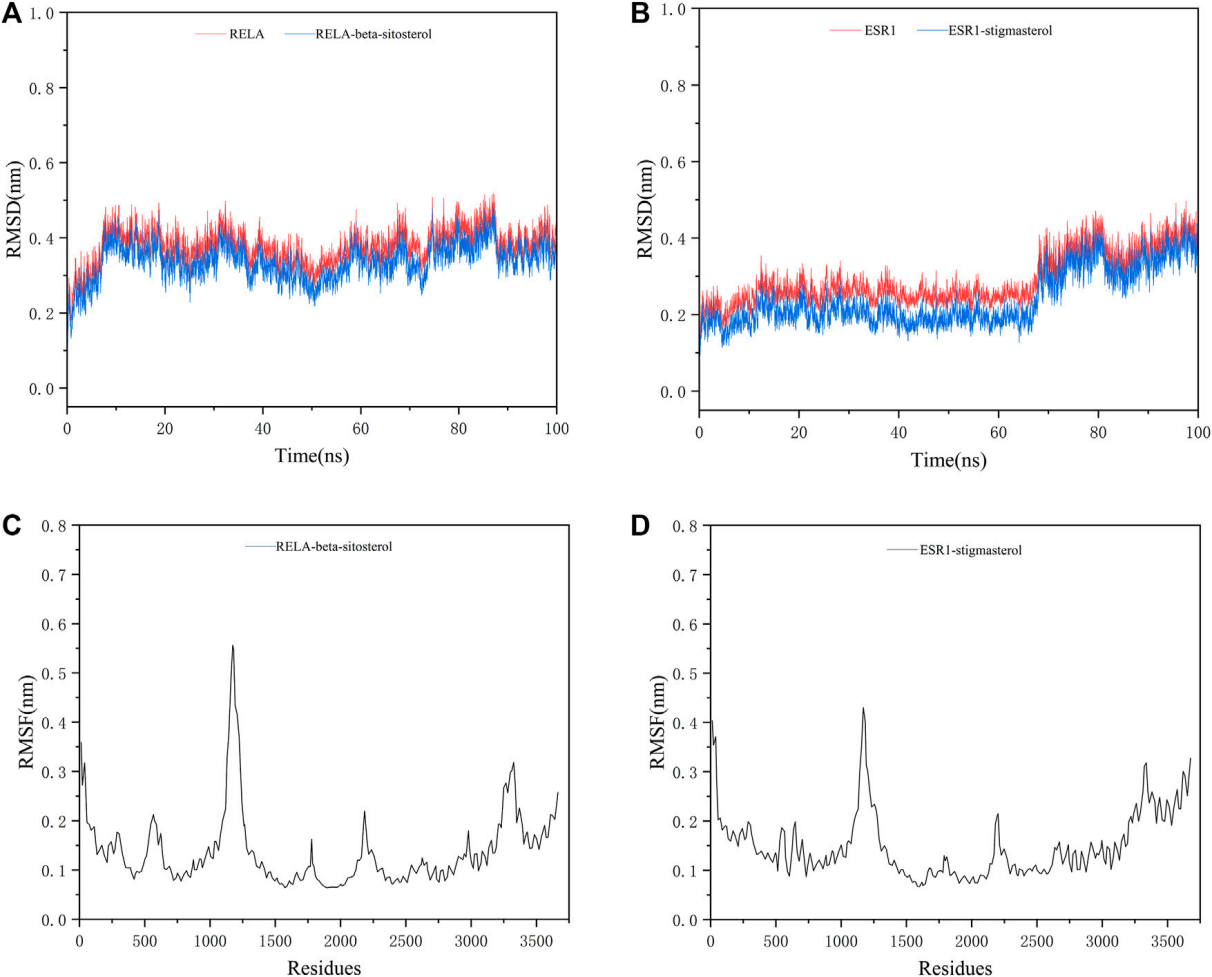
The MD simulation results of two complexes. **(A)** The RMSD analysis for the RELA-beta-sitosterol complex. **(B)** The RMSD analysis for the ESR1-stigmasterol complex. **(C)** The RMSF analysis for the RELA-beta-sitosterol complex. **(D)** The RMSF analysis for the ESR1-stigmasterol complex.

We selected the top two ingredient-target dockings (RELA-beta-sitosterol and ESR1-stigmasterol) to conduct molecular dynamics simulations. After 100 ns of MD simulations, the dynamic evolutions of the RELA-beta-sitosterol and ESR1-stigmasterol complexes could be analyzed. The RMSD curve represents positional deviations in the protein. As can be seen from Figures 13A, B, the RELA-beta-sitosterol complex was in an equilibrium state and the average RMSD value was 0.34448675 during 80–100 ns. Meanwhile, the ESR1-stigmasterol complex had a rise within 90 ns and tended to balance out the last 10 ns with the average RMSD value was 0.35742059. The low fluctuation of the two complexes indicated that their stability was stronger. The RMSF curve represents the fluctuation of the protein amino acid residues. The fluctuations of the two complexes in the 1,108–1,203 regions were relatively high. Inversely, most of the residues fluctuated at lower values in other regions, suggesting that the binding residues of RELA-beta-sitosterol and ESR1-stigmasterol were stable (Figures 13C, D). In addition, the binding energy of RELA-beta-sitosterol was −129.870 kcal/mol, and the ESR1-stigmasterol complex was −121.686 kcal/mol (Table 4).

## 4 Discussion

COPD is a chronic inflammatory airway disease that can be prevented and treated. In recent 10 years, the prevalence of COPD in Chinese over 40 years old has increased from 8.2% to 13.7%, and the risk of hospitalization and mortality has also increased (Zhang et al., 2019; Zhang et al., 2019). Notably, TCM treatment can relieve the symptoms of COPD patients and improve their survival rate. As classical formulas of TCM in COPD treatment, ECXB formula has respectively obtained some achievements in clinical efficacy and action of mechanism. However, the study of the combination of the two formulas for COPD is still in the initial stage. In this study, we used the SymMap platform to collect related symptoms treated by both TCM and modern medicine of herbs in the formula of ECXB to conduct Herb-Symptom comparative analysis, and applied the network pharmacology, molecular docking, and MD simulation to explore the potential mechanisms of ECXB formula on COPD in order to provide theoretical basis for the following validation studies.

Firstly, we comparative analysis Herb-Symptom network symptoms targeted by TCM and modern medicine as well as COPD symptoms, and found that the symptoms treated by both TCM and modern medicine of herbs in the formula of ECXB were similar to the symptoms of COPD patients, indicating that ECXB formula treatment of COPD conforms to the symptomatic treatment principle of TCM. Then we analyzed the “Drug-ingredient-target” network, *hederagenin* and *naringenin* (the main ingredients of *Citri reticulatae pericarpium* and *Poria* in Erchen Decoction), and *kaempferol* and *hederagenin* (the main ingredients of *Mori cortex* and *Lycii cortex* in Xiebai Powder), are the core active ingredients of ECXB formula for COPD treatment. *Hederagenin* is a pentacyclic triterpenoid compound isolated from plants, which has been shown to have strong antibacterial, anti-inflammatory, and anti-tumor biological activities (Ndjateu et al., 2014; Fang et al., 2018). Notably, it can reduce the level of proinflammatory cytokines in serum and lung tissue and reduce the damage of oxidative stress response to the lung (Wang and Zhao, 2022). *Naringenin* has anti-inflammatory, scavenging free radicals, inhibiting peroxidation, lowering blood lipids, and inhibiting tumor growth (Annadurai et al., 2013; Alam et al., 2014), and it is thought to be beneficial in COPD due to its anti-inflammatory properties (Chin et al., 2020). Liu et al. (2018) study indicated that *naringenin* attenuates inflammation in COPD in cigarette smoke induced mouse model and involves suppression of NF-κB, and they concluded that it can be a potential therapeutic agent for the treatment of COPD-related inflammation. *Kaempferol* has antioxidant stress activity and anti-inflammatory activity, which significantly inhibits the expression of the MAPK pathway, reduces the production of various inflammatory factors, and effectively inhibits inflammation (Park et al., 2006; Alam et al., 2014). Tsou et al. (2014) study found that *kaempferol* was found to be IKK2 inhibitor and helped prevent COPD occurrence and worsening. In summary, it can be inferred that the core ingredients of ECXB formula play a therapeutic role by affecting inflammatory factors related to COPD.

PPI network analysis results suggest that the core targets of ECXB formula for the treatment of COPD mainly include MAPK8, ESR1, TP53, MAPK3, JUN, RELA, MAPK1, and AKT1. Among them, MAPK8, MAPK3, and MAPK1 are intracellular serine/threonine protein kinases, which can change gene expression by phosphorylation of transcription factors, promote the production of inflammatory factors TNF-α, IL-1, IL-6, and induce a series of inflammatory and immune responses, thus playing a role in the pathogenesis of COPD (Kang et al., 2013; Duan et al., 2020). TP53 is a tumor suppressor that plays a variety of roles in controlling cell cycle checkpoints, apoptosis, and DNA repair. In COPD smokers, downregulation of TP53 - and p53-related signal transduction may lead to lung tumors (Zhou et al., 2021). ESR1 gene can code estrogen receptors, and studies have found that estrogen signaling may play a key role in lung diseases. Overexpression of ESR1 m RNA is closely related to the prognosis of non-small cell lung cancer (Suga et al., 2008), while ESR1 is expressed in lung tissues of patients with COPD, but its specific mechanism remains unclear. JUN is the most widely used protein in activating protein 1 complex, (Bin et al. (2020) discovered that c-Jun is a potential novel therapeutic target for COPD, JNK inhibition by erythromycin restores corticosteroid sensitivity via the inhibition of c-Jun expression. AKT1 is an important factor in the PI3K-AKT signaling pathway, which plays a key role in many physiological processes such as cell growth and survival and is associated with airway inflammation and lung function changes in asthma (gianni, 2019). In conclusion, the core targets may influence the onset and progression of COPD through inflammatory immunity, reducing inflammatory factors and oxidation-reduction reaction, and so on.

We also performed GO function and KEGG pathway enrichment analyses. Response to drug, response to nutrient levels, and response to extracellular stimulation may be the key biological processes in the ECXB formula treatment of COPD (Bidan et al., 2015; Beijers et al., 2022). Membrane raft and amide binding were the most significant cellular component and molecular functions, respectively. It has been reported that preponderance of saturated fatty acids in plasma membrane of erythrocytes of COPD patients which may decrease the membrane fluidity and possibly impair the functions of the plasma membrane in the disease (Gangopadhyay et al., 2012). According to the KEGG analysis, the TNF signaling pathway, cAMP signaling pathway, and VEGF signaling pathway were considered key pathways in the treatment of COPD by ECXB formula. The TNF pathway is critical in inflammatory response by regulating both apoptosis and proliferation. Lee et al. (2018) display that fisetin is a good therapeutic drug for the treatment of inflammatory lung diseases, such as COPD, by inhibiting the TNF-alpha/NF-kappa B signaling pathway. Arora et al. (2022b) discovered that a significant reduction in TNF-α levels and concomitant suppression of asthma symptoms after oral administration of solasodine in ovalbumin-sensitized rats are indicative of the possible therapeutic role of phytocompound in chronic asthma irresponsive to ICS. Liu et al. (2015) study results suggested that IL-15 inhibited protein degradation in skeletal muscle in COPD rats, which may be mediated by the TNF-alpha and UPP pathway. Arora et al. (2021) and Arora et al. (2022a) studies demonstrate that biomarkers of the inflammatory response, including cell count, immunoglobulin E, cytokines such as interleukin (IL-4), −5, −1β, tumor necrosis factor (TNF)-α, etc. The findings that Mesua ferrea L. and Clerodendrum serratum in allergic asthma may be related to the ability of the plant to attenuate inflammatory cell responses and thus to the production of inflammatory and pro-inflammatory cytokines in the airways. The cAMP pathway plays a major role in COPD signaling. For instance, Dunne et al. (2019) study showed that roflumilast inhibits neutrophil chemotaxis directly via a cAMP-mediated mechanism requiring activation of Epac1 and that Epac1 activators could reduce COPD neutrophilic inflammation. Nabissi et al. (2018) work demonstrated that thyme extract is effective in stimulating CBF by inducing an increase of cAMP and Ca^2+^ levels, thus supporting its therapeutical use in the treatment of COPD. The VEGF signaling pathway has been related to the pathogenesis of COPD. Zhang et al. (2022) the efficacy of electroacupuncture (EA) with respect to the regulation of microvascular remodeling induced by VEGF/PI3K/Akt was evaluated in a rat model of COPD, showed that reduced pulmonary vascular remodeling via mechanisms possibly related to the VEGF/PI3K/Akt pathway. Willems-Widyastuti et al. (2011) study suggested that TGF-beta (1) could play a key role in bronchial angiogenesis and vascular remodeling via VEGF pathway in asthma. The active ingredients of ECXB formula may act on these signaling pathways in the treatment of COPD.

The network pharmacology results were verified for ECXB formula in the treatment of COPD by the molecular docking of the 8 core targets and the top ten selected active ingredients. The results of the molecular docking indicated that the core active ingredients of ECXB formula in the treatment of COPD could bind well with their core targets. Among them, RELA-beta-sitosterol and ESR1-stigmasterol had the lowest binding scores. MD simulation was employed to further verify the results obtained. The results of molecular dynamics simulation suggested that RELA-beta-sitosterol and ESR1-stigmasterol could bind tightly, and the binding energies of the complexes were −129.870 kcal/mol, and −121.686 kcal/mol, respectively.

However, it should be noted that there were certain limitations of the network pharmacology. The study based on network pharmacology is a static network analysis while the occurrence and development of disease and the action of drugs are both dynamic processes. Therefore, the following experiments *in vivo* or *in vitro* can be carried out on this basis to explore the deeper mechanism of ECXB formula in the treatment of COPD. Despite the limitations of this study, the results revealed that it had certain clinical value and research significance.

## 5 Conclusion

Altogether, we analyzed the role of ECXB formula in treating COPD from the Herb-Symptom network relationship and utilized network pharmacology, molecular docking, and MD simulation to explore the potential mechanisms of ECXB formula in treating COPD. The results indicated that hederagenin, naringenin, kaempferol, and other effective ingredients of ECXB formula showed therapeutic effects against COPD via multiple targets and multiple pathways. In addition, the good activities of the core active ingredients and the core targets were verified by molecular docking and MD simulation. In summary, the results of this study provided a reference for clinical application and guidance for further experimental verification of ECXB formula for the treatment of COPD.

## Data Availability

The original contributions presented in the study are included in the article/Supplementary Material, further inquiries can be directed to the corresponding authors.
